# Compound heterozygous variants in DYNC2H1 in a foetus with type III short rib-polydactyly syndrome and situs inversus totalis

**DOI:** 10.1186/s12920-022-01205-z

**Published:** 2022-03-12

**Authors:** Chen Cheng, Xiuxiu Li, Sheng Zhao, Qian Feng, Xiang Ren, Xinlin Chen

**Affiliations:** 1 Department of Ultrasonography, Maternal and Child Health Hospital of Hubei Province, Tongji Medical College, Huazhong University of Science and Technology, Wuhan, 430070 China; 2 College of Life Science and Technology, Huazhong University of Science and Technology, Wuhan, China

## Abstract

**Background:**

Short-rib thoracic dysplasia 3 with or without polydactyly (SRTD3, OMIM: 613091) is an autosomal recessive disorder. SRTD3 presents clinically with a narrow thorax, short ribs, shortened tubular bones, and acetabular roof abnormalities. Clinical signs of SRTD3 vary among individuals. Pathogenic variants of *DYNC2H1* (OMIM: 603297) have been reported to cause SRTD3.

**Methods:**

We performed a detailed clinical prenatal sonographic characterization of a foetus with SRTD3. Trio whole-exome sequencing was used to identify causative variants in the family. The identified variants in the families were validated by Sanger sequencing and mass spectrometry. Multiple computational tools were used to predict the harmfulness of the two variants. A minigene splicing assay was carried out to evaluate the impact of the splice-site variant.

**Results:**

We evaluated prenatal sonographic images of the foetus with SRTD3, including abnormal rib curvature, narrow thorax, bilateral hypoplastic lungs, bilateral polydactyly, syndactyly, and foetal visceral situs inversus with mirror-image dextrocardia. We revealed novel compound variants of *DYNC2H1* (NM_001377.3:c.11483T > G (p.Ile3828Arg) and c.2106 + 3A > T). Various statistical methods predicted that the variants would cause harmful effects on genes or gene products. The minigene assay findings suggested that c.2106 + 3A > T caused the skipping over exon 14, producing an exon 14 loss in the protein.

**Conclusion:**

This study identified a foetus with SRTD3 with situs inversus totalis with mirror-image dextrocardia in a Chinese family, revealing two novel compound heterozygous dynein cytoplasmic 2 heavy chain 1 (*DYNC2H1*) variants, expanding the phenotypic spectrum of SRTD3. The minigene study of c.2106 + 3A > T was predicted to cause an inframe exclusion of exon 14, which was predicted to have important molecular functions. Our findings strongly supported the use of WES in prenatal diagnosis and helped to understand the correlation of genotype and phenotypes of *DYNC2H1*. The specific sonographic findings and the molecular diagnosis helped add experience to further our expertise in prenatal counselling for SRTD3.

**Supplementary Information:**

The online version contains supplementary material available at 10.1186/s12920-022-01205-z.

## Introduction

Short-rib thoracic dysplasia 3 with or without polydactyly (SRTD3, OMIM: 613091) is an autosomal recessive disorder. SRTD3 presents clinically with a narrow thorax, short ribs, shortened tubular bones, and acetabular roof abnormalities [1]. Clinical signs of SRTD3 vary among individuals. Nonskeletal manifestations also include cleft lip and palate and some major organs, such as the brain, eyes, heart, kidneys, liver, pancreas, intestinal abnormalities, and genitalia [2]. Some foetuses die in the neonatal period due to severe restriction of the thorax secondary to respiratory insufficiency, while others survive. There are many SRTD subtypes. The four lethal SRTDs are SRTD types I-IV. SRTD types I-IV are mainly characterized by short limb deformities, thoracic stenosis, often combined with polydactyly and multiple anomalies of other organs. Each SRTD subtype has its typical features and a common overlapping phenotype [3]. The prenatal phenotype differs and has variable expressivity. Therefore, it is a challenge to provide a timely and accurate diagnosis [4]. Sonographic examinations provide clues, and genetic tests help to give a molecular diagnosis.

## Methods

### Patient and sonographic examinations

A 22-year-old woman was referred to our department for routine sonographic examination at 22.4 gestational weeks (GW). Systematic prenatal sonographic examinations, including two- and three-dimensional ultrasound and echocardiography, were obtained to a final diagnosis by at least two chief physicians. GE Volusion E10 was used to perform the sonographic examination with a 1–5 MHz probe. These sonographic scans were all performed using a GE Volusion E10 machine with a 1–5 MHz sector transducer.

### Genetic testing

Trio whole-exome sequencing (WES) of the family was performed at BGI company, Wuhan, China. In this method, genomic DNA was collected from blood from the parents and umbilical cord blood from the foetus. First, DNA was broken into 100- to 500-bp fragments. The 200- to 300-bp fragments were collected by magnetic beads (VAHTS™ DNA Clean Beads, Vazyme). Base “A” was added at the 3' overhangs, which could pair the “T” base with a special adapter. After elution and postcapture amplification, the library was enriched for 16 h at 65 °C by array hybridization (KAPA Hyper Exome, Roche). The magnitude of enrichment of the product was estimated. The qualified products were pooled, quantified, and prepared for circularization. Finally, the MGISEQ-2000 sequencing platform was used for variant detection. The average sequencing depth of the target region was ≥ 180X, and the percentage of loci with an average depth of > 20X in the target region was > 95%. After the sequencing was completed, raw reads from the machine were evaluated for sequencing quality, and low-quality and joint-contaminated reads were removed for further analysis [5].

### Data analysis

Sequenced fragments were aligned with the UCSC hg19 human reference genome by BWA (Burrows Wheeler Aligner) to remove duplicates [6]. Base quality score recalibration, SNVs (single-nucleotide variants), InDels (insertions and deletions), and genotypes were detected using GATK software (GATK version number 4.1.9) [7]. Variation annotation and screening were conducted based on the clinical information of subjects, population databases, disease databases, and biological information prediction tools, including NCBI dbSNP, HapMap, the 1000Genomes human dataset, and a database of 100 Chinese healthy adults. ExomeDepth was used for copy number variation (CNV) detection at the exon level. The variants were named according to the Human Genome Variation Society (HGVS) nomenclature. To predict the CNV, we used the in-house database (https://phoenix.bgi.com/autocnv/). To predict the effects of missense variants, we used dbNSFP, which contains seven well-established in silico prediction programs (Scale-Invariant Feature Transform (SIFT), Polyphen2, LRT, Mutation Taster, and PhyloP) [8]. To predict the effect of splice variants, we used prediction tools (SpliceAI, dbscSNV_ADA and dbscSNV_RF). To predict the conservation of variants, we used tools such as PhyloP Vertebrates, PhyloP Placental Mammals and GERP++_RS. Variation pathogenicity was classified according to the American College of Medical Genetics and Genomics (ACMG), the Association for Molecular Pathology (AMP) Sequence Variation Interpretation Guidelines, the Clingen Sequence Variation Interpretation Working Group and the Association for Clinical Genomic Science (ACGS), and other detailed interpretations of these guidelines [9].

### Variant validation

To verify the results of gene chip capture and high throughput sequencing, the two suspected variants were validated by Sanger sequence and mass spectrometry. The *DYNC2H1* variant c.2106 + 3A > T (NM_001377.3) was validated by Sanger sequencing. Primers were designed upstream and downstream of the segment. Polymerase chain reaction (PCR) amplification and Sanger sequencing were performed using the forward primer TTTGCCAGACTGCCTTATCA and the reverse primer AGACTGAGGAGGGGAGGCTA. The *DYNC2H1* c.11483T > G (NM_001377.3) validation was performed by using the MassARRAY system (Agena Bioscience company) according to a previously described method [10]. The MassARRAY System uses matrix-assisted laser desorption/ionization-time of flight (MALDI-TOF) mass spectrometry for the precise detection of DNA molecules. Genetic variants are distinguished by analysis of their mass. The probe sequence was CTACAGTCAAGTCTGAAGA.

### Minigene splicing assay

A genomic segment encompassing the variant c.2106 + 3A > T sequence along with flanking intronic sequences, including exon 13, part of intron 13–14, exon 14, intron 14–15 and exon 15, was PCR-amplified from patient genomic DNA. The segments containing the wild-type (WT, c.2106 + 3A) and mutant-type (MT, c.2106 + 3T) sequences were separately cloned into the minigene vector pMini-CopGFP (Hitrobio.tech, China). The following primers were used to construct the vectors using the ClonExpress® II One Step Cloning Kit (Vazyme Biotech Co., Ltd): *DYNC2H1*-AF, AAGCTTGGTACCGAGCTCGGATCCGTGGCACATTTTTATAATTCTATTGATC; *DYNC2H1*-AR, AAGAAGGCAGACAGCCATGGGGTTTCACCATGTTGG; *DYNC2H1*-BF, ATGGCTGTCTGCCTTCTTCATTCTTCCTTTCCG; *DYNC2H1*-BR, TTAAACGGGCCCTCTAGACTCGAGCTGTGCTTCTACAGTTGCTAAGCCAGTT. The correct wild-type and mutant Minigene plasmids were transfected into HEK293T cell line for 48 h. Total RNA was extracted, and cDNA synthesis was performed (R212-01, Vazyme Biotech Co., Ltd.). Real-time PCR was performed using the primers F-GGCTAACTAGAGAACCCACTGCTTA and R-CTGTGCTTCTACAGTTGCTAAGC, and the products were sequenced.

## Results

### Patient and sonographic findings

**Fig. 1:**
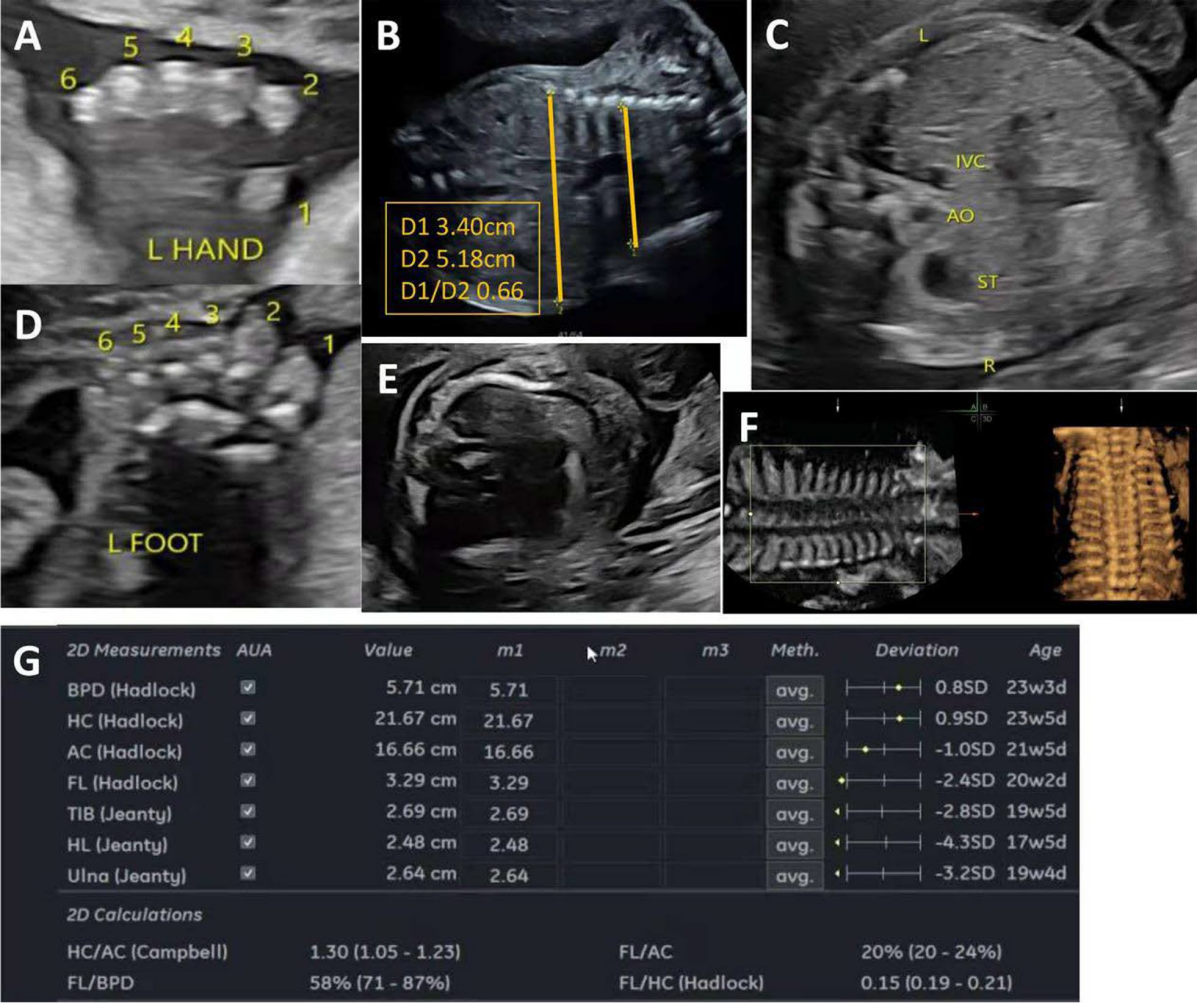
The sonographic results of the foetus. **A**, **D** Polydactyly, and syndactyly. **B** The thoracoabdominal transverse diameter ratio was 0.66. **C** The abdominal organs of the foetus were in the opposite position of normal, i.e., in a mirror image position, with the gastric vacuole and descending aorta on the right, the apex of the heart facing right, and the gallbladder, and inferior vena cava (IVC) located on the left side. **E**, **F**: Abnormal rib curvature and narrow thorax. **G** Ultrasound measurements (Hadlock and Jeanty) based on gestational age. The FL, TIB, HL and ulnar values were below 2.4 SD, 2.8 SD, 4.3 SD and 3.2 SD, respectively, compared with the average. Abbreviations: L, left; R, right; IVC, inferior vena cava; AO, aorta; ST, stomach; BPD, biparietal diameter; HC, head circumference; AC, abdominal circumference; FL, femur length; TIB, tibia length; HL, humerus length

A 22-year-old woman was referred to our department for routine sonographic examination at an ultrasonic gestational age of 20.6 weeks and 22.4 weeks from the last menstrual period (LMP). This was the second pregnancy of the woman. She had an early pregnancy loss in her first pregnancy. The couple was healthy and non-consanguineous. The sonographic examination detected multiple anomalies. The measurement value (− 2 standard deviation (SD)/+ 2 SD) of the biparietal diameter (BPD) was 5.7 cm (4.9 cm/6.1 cm); the head circumference (HC) was 21.7 cm (18.3 cm/21.8 cm); the abdominal circumference (AC) was 16.7 cm (15.7 cm/19.6 cm), the femur length (FL) was 3.3 cm (3.3 cm/4.1 cm); and the humerus length (HL) was 2.5 cm (3.21 cm/4.05 cm), respectively. (Fig. 1) Both the HL and FL values were below – 2 SD. The foetus showed abnormal rib curvature, a narrow thorax, bilateral hypoplastic lungs, bilateral polydactyly, a hallux valgus angle on the foot and syndactyly. The foetus was situs inversus totalis with mirror-image dextrocardia. The liver and gallbladder inferior vena cava was on the left side of the body, the stomach and aorta were on the right, and the spleen was on the right, but the spleen was not easy to visualize in the foetus. The heart presented with mirror-image dextrocardia. The bilateral humerus was curved (Fig. 1).

An equal echo of approximately 0.4*0.3 cm in size was seen on the lateral aspect of the little fingers on both sides. A strong osseous echo could be seen inside. The ribs were short and curved. The thoracoabdominal junction was markedly depressed, and the thorax was markedly reduced (3.4/5.18 = 0.66). The thoracoabdominal transverse diameter ratio was 0.66. The size of the left lung was 2.7 * 1.1 cm, and that of the right lung was 1.6 * 1.0 cm. The two foetal kidney collecting systems were separated by approximately 0.4 cm. Echoes of 6 toes were seen on the left foot. The first and second toes of the right foot were widely spaced, and the fourth and fifth toes appeared to have syndactyly. The tibial length was 2.7 cm (− 2.8 SD); the ulnar length was 2.6 cm (− 3.2 SD). The final US diagnosis was short-rib thoracic dysplasia (narrow thorax, broken ribs with curvature, short limb deformities, polydactyly, hallux valgus angle), situs inversus totalis, and mirror-image dextrocardia. The parents chose to terminate the pregnancy due to multiple anomalies.

### Genetic findings

**Fig. 2:**
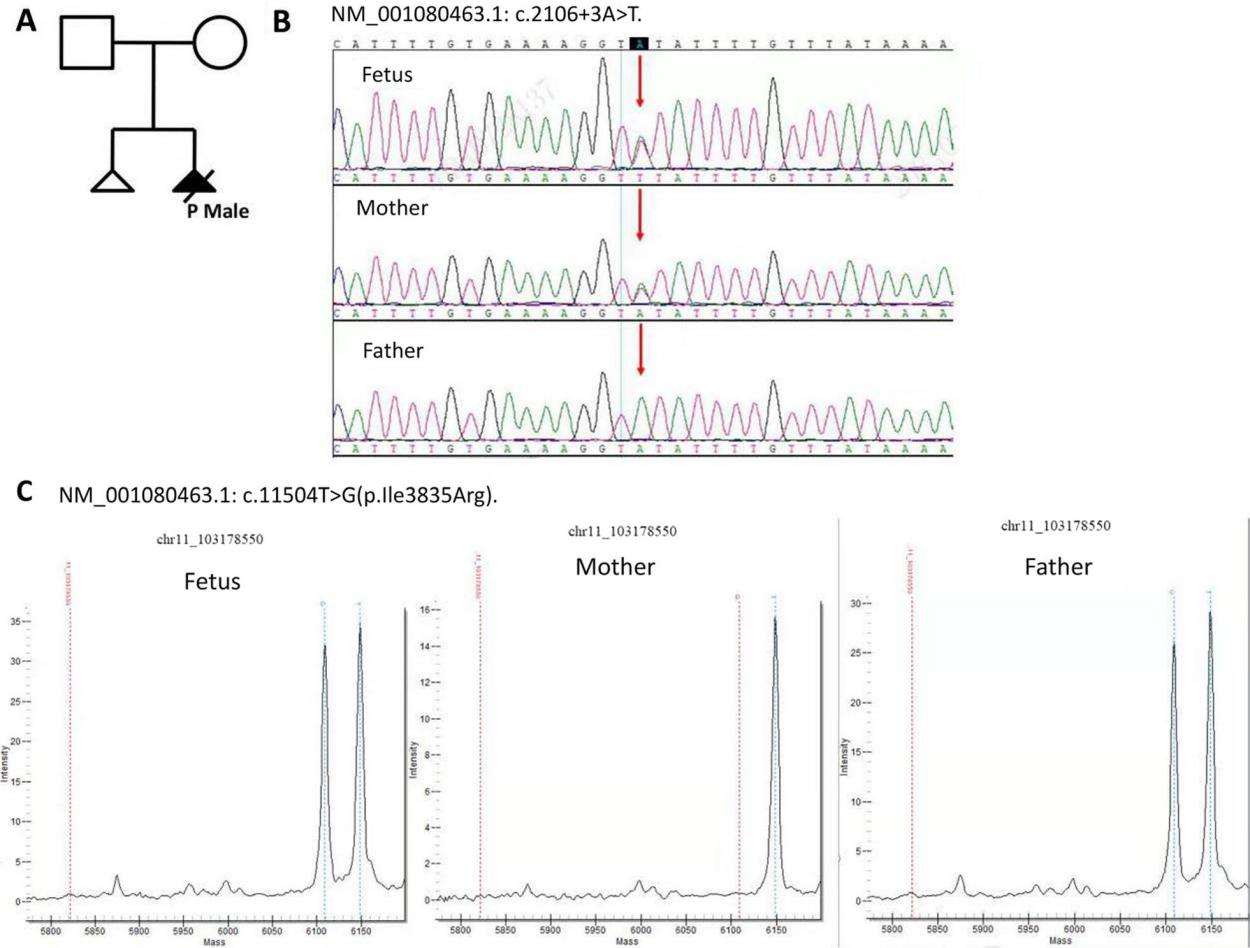
Compound heterozygous variants in *DYNC2H1* in a family with SRTD3. **A** Familial pedigree. **B** *DYNC2H1* NM_001377.3: c.11483 T > G was confirmed in family members by Sanger sequencing, and the foetus carried the c.11483G inherited from the mother. **C** *DYNC2H1* NM_001377.3 c.2106 + 3A > T was confirmed by mass spectrometry, and the foetus carried the c.2106 + 3 T inherited from the father

Two heterozygous variants of the *DYNC2H1* gene associated with the subject's phenotype in SRTD3 were detected, NM_001377.3: c.11483T > G (p.Ile3828Arg) and c.2106 + 3A > T. We did not detect rare or likely pathogenic homozygous or heterozygous variants in other STRD-causing genes in family trio WES. No pathogenic chromosomal CNV variants above 1 M and LOH variants above 5 M associated with the phenotypes of the proband were detected through the analysis of the high-throughput data. c.11483T > G (p.Ile3828Arg) was detected in the father and the foetus in family trio WES. The c.11483T > G variant was located in exon 78 of the *DYNC2H1* gene and caused the alteration of isoleucine to arginine at amino acid position 3828. c.11483T > G had frequencies of 0.000013 in GnomAD and 0.00019 in GnomAD-EAS (East Asian population). This variant was not found in the control population in the ESP, 1000, and EXAC databases. Various statistical methods, including conservative and evolutionary predictions, predicted that the variant would cause harmful effects on genes or gene products. Computer software tools, including SIFT, Mutation Taster, and Condel, predicted that predicted the missense variant was pathogenic. All three tools (PhyloP Vertebrates, PhyloP Placental Mammals, GERP++) showed that c.11483T > G was conserved (Table 1). c.2106 + 3A > T was detected in the mother and the foetus in family trio WES. No frequency was found in the ESP, 1000, EXAC, and GnomAD databases. All three tools predicting splicing site effects (SpliceAI, dbscSNV_RF, dbscSNV_ADA) raised the possibility that c.2106 + 3A > T caused loss of function for authentic splice sites (Table 2). The SpliceAI showed that the probability that position 11:103,004,436 (= 103,004,439–3) was used as a splice donor was decreased by 0.72. Mass spectrometry confirmed that the foetus carried c.11483  > G, which was inherited from the father. Sanger sequencing confirmed that the foetus carried c.2106 + 3A > T, which was inherited from the mother (Fig. 2).

**Table 1:** Conservation prediction of the two variants by PhyloP vertebrates, PhyloP placental mammals, and GERP++

HGVS-c	PhyloP vertebrates	PhyloP placental mammals	GERP + + _RS
c.2106 + 3A > T	0.767	2.168	
c.11483 T > G	4.426	2.118	5.53

**Table 2:** Functional prediction of the two variants by SpliceAI, dbscSNV_RF, and dbscSNV_ADA algorithms

HGVS_c	SpliceAI	SpliceAI Pred	SpliceAI Interpretation	dbscSNV_ADA_SCORE	dbscSNV_ADA_pred	dbscSNV_RF_SCORE	dbscSNV_RF_pred
c.2106 + 3A > T	SpliceAI = T|DYNC2H1|0.00|0.00|0.00|0.72|13|-3|-11|-3	D	11:103,004,436 (= 103,004,439–3) donor loss 0.72	0.9994	D	0.93	D
c.11483 T > G	SpliceAI = G|DYNC2H1|0.04|0.00|0.02|0.00|-48|-8|10|-2	P

### Splicing study of the *DYNC2H1* c.2106 + 3A > T variant

**Fig. 3:**
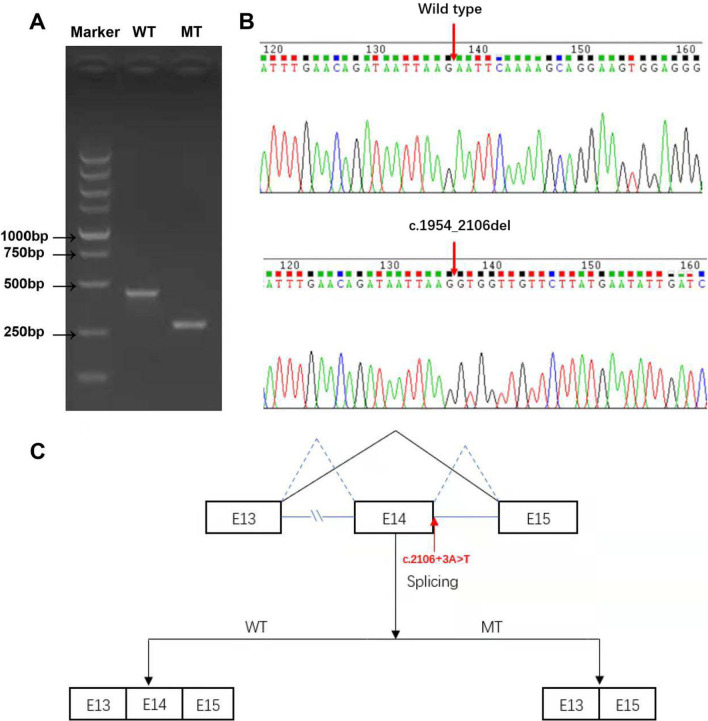
Minigene splicing study of the c.2106 + 3A > T. After transfection with WT and MT minigene plasmids in the HEK293T cell line, total RNA was extracted and cDNA was synthesized. The target fragment was amplified by RT–PCR. **A** Agarose gel electrophoresis results of the RT–PCR products; the target fragment was shorter in MT than in WT. **B** Sanger sequencing of the RT–PCR products; MT caused c.1945_2106del, indicating the loss of the whole exon 14. **C** Schematic diagram showing the aberrant splicing

A minigene expression assay revealed the synthesis of a messenger RNA lacking exon 14, which generated a predicted in-frame deleted protein. The agarose gel electrophoresis result is shown in Fig. 3A. The predicted PCR amplification of the WT sequence length was 419 bp, while the MT sequence length was 266 bp, lacking the 153-bp exon 14. Sanger sequencing of the RT-PCR products confirmed that MT caused mRNA skipping over exon 14 (Fig. 3B). We have drawn a schematic diagram to illustrate aberrant splicing, as shown in Fig. 3C.

## Discussion

Many genes have been reported to be related to SRTD3, such as *DYNC2H1*, *DYNC2LI1*, *WDR19*, *WDR34*, *WDR35*, *WDR60*, *IFT140*, *TTC21B, TCTEX1D2*, *IFT80*, *IFT172*, *EVC1*, *EVC2*, *KIAA0586*, *CEP120, INTU, ICK, NEK1*, and *C21ORF2* [11]*.* Among the above genes, *DYNC2H1* is the most common gene associated with SRTD3 disease. *DYNC2H1* encodes a dynein protein that participates in ciliary intraflagellar transport [12]. Mutations in *DYNC2H1* will lead to the dysfunction of primary cilia, causing a heterogeneous spectrum of conditions such as skeletal dysplasias [13]. *DYNC2H1* is the pathogenic gene of SRTD3 disease. SRTD3 disease is clinically and genetically heterogeneous. There is significant variability in the course and severity of the thoracic phenotype, both between siblings with the same *DYNC2H1* allele and between individuals with different alleles, suggesting that the *DYNC2H1* phenotype may be influenced by modified alleles and nongenetic or epigenetic factors [14]. More details about how each variant causes its phenotype await further study of the mechanisms of gene function.

SRTD3 generally presents prenatally with extremely shortened long bones and ribs, round metaphyseal ends and lateral spikes, a small and narrow thorax, and preaxial and postaxial polydactyly, sometimes presenting with bowing femora, genitals and multiple malformations [15]. Among all the cases of reported SRTD, Okamoto et al. once reported a Japanese patient carrying a c.5682_5683 delAA and c.9010C > T mutations who showed an extremely severe phenotype, including rib and long bone shortness, severe thoracic hypoplasia, polydactyly, ventriculomegaly, macrocephaly, polyhydramnios, visceral heterotaxia, a congenital heart defect and hypoplasia of the iliac bones [16]. Baujat et al. reported a baby in Turkey aged 9 months who was diagnosed with SRTD3 with situs inversus [17]. As evidenced by this study of SRTD3 with situs inversus totalis and mirror-image dextrocardia at the prenatal stage, we added one more detailed piece of the situs inversus phenotype with SRTD3 in a Chinese foetus.

The *DYNC2H1* compound heterozygous variants of the autosomal recessive inheritance pattern were consistent with SRTD3 disease. The foetal manifestation accorded with the SRTD3. No rare or likely pathogenic homozygous or heterozygous variant in other STRD-causing genes was found in family trio WES. We performed a minigene splicing study and confirmed that the variant caused a skipping over exon 14. Though no pathogenic variants were reported in this region previously, exon 14 was predicted to participate in many GO processes such as molecular_function, protein binding, catalytic activity and so on by NetGO 2.0 [18]. According to the ACMG/AMP guidelines, c.11483T > G (p.Ile3828Arg) was classified as Variants of Uncertain Significance with the criteria PM2 and PP3 and c.2106 + 3A > T was classified as Likely Pathogenic with the criteria PM2, PP3 and PS3. On all these counts, the compound heterozygous variants in *DYNC2H1* are highly likely the cause of the SRTD3 with polydactyly in this patient.

Here we presented a foetus with abnormal rib curvature, narrow thorax, bilateral hypoplastic lungs, bilateral polydactyly, syndactyly and foetal visceral situs inversus with mirror-image dextrocardia presentations caused by compound heterozygous variants in *DYNC2H1*. These findings expanded the spectrum of *DYNC2H1* variants which was more diverse and predicted important molecular functions of exon 14 of *DYNC2H1*. The specific sonographic findings and the molecular diagnosis helped add experience to further our expertise in prenatal counselling for SRTD3. Our findings strongly supported the use of WES in prenatal diagnosis and further helped to understand the correlation of genotype and phenotypes of *DYNC2H1* variants. Next-generation sequencing was an effective method of prenatal diagnosis of a genetic skeletal disorder.

## Supplementary Information

**Additional file 1: Figure S1**. The original image of figure 3A.

## Data Availability

The novel variants revealed during the study were submitted to ClinVar database (https://www.ncbi.nlm.nih.gov/clinvar/) under Accession Numbers SCV001806774 and SCV001806775.
