# Supplementary figures and images for: Compound heterozygous variants in DYNC2H1 in a foetus with type III short rib-polydactyly syndrome and situs inversus totalis
Source: BMC Med Genomics. 2022 Mar 12;15:55. doi: 10.1186/s12920-022-01205-z (PMC8917749; doi:10.1186/s12920-022-01205-z)

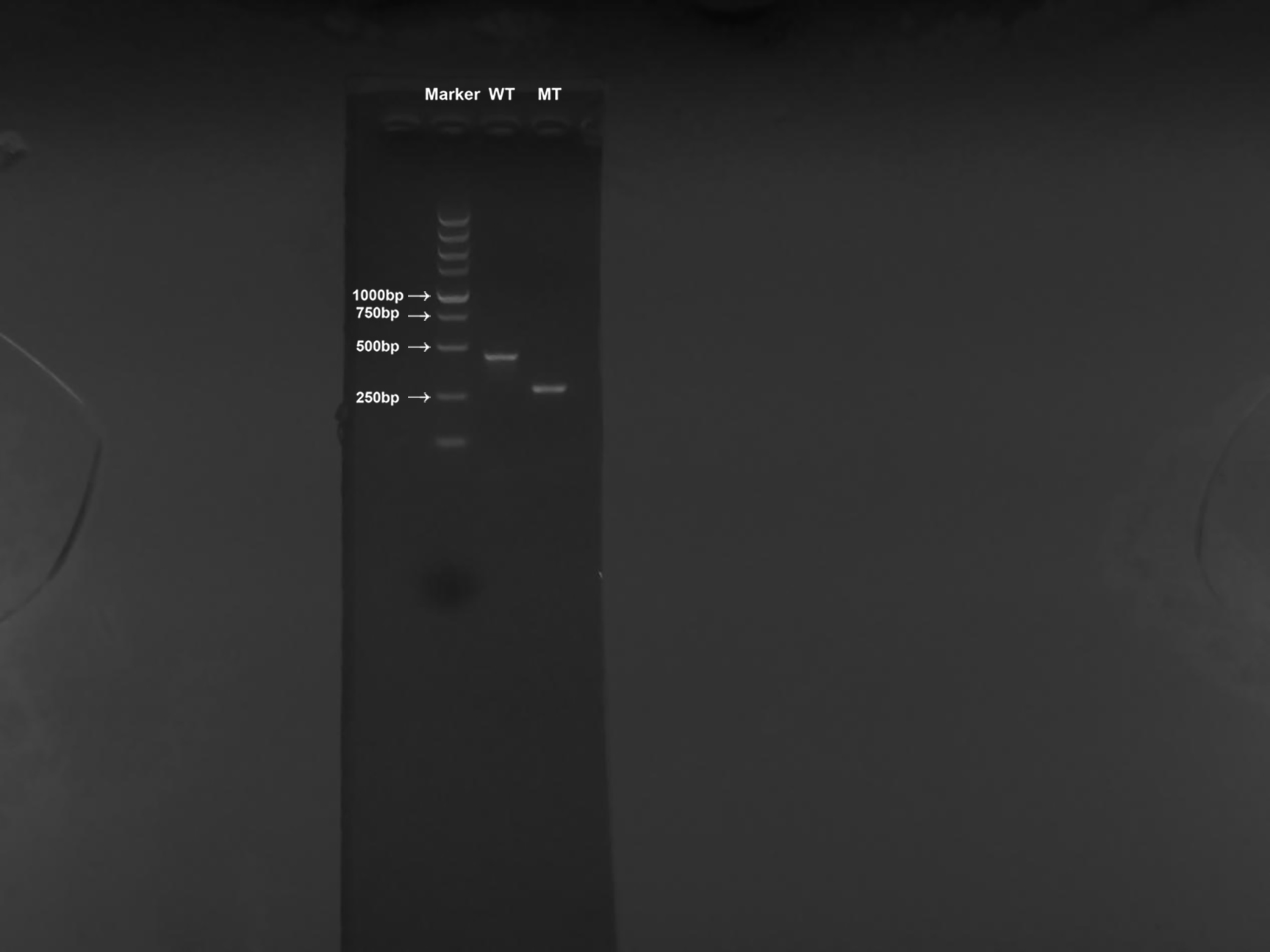

Supplement: Supplementary file 1 — Additional file 1: Figure S1. The original image of figure 3A. [file 12920_2022_1205_MOESM1_ESM.jpg]
